# Effect of ulixertinib, a novel ERK1/2 inhibitor, on the QT/QTc interval in patients with advanced solid tumor malignancies

**DOI:** 10.1007/s00280-018-3564-1

**Published:** 2018-03-30

**Authors:** Boaz Mendzelevski, Georg Ferber, Filip Janku, Bob T. Li, Ryan J. Sullivan, Dean Welsch, Wei Chi, Jeanne Jackson, Onglee Weng, Philip T. Sager

**Affiliations:** 1 Cardiac Safety Consultants Ltd, 4 Hallswelle Road, London, NW11 0DJ UK; 2 Statistik Georg Ferber GmbH, Riehen, Switzerland; 3 The University of Texas MD Anderson Cancer Center, Houston, TX USA; 4 Memorial Sloan Kettering Cancer Center, New York, NY USA; 5 Massachusetts General Hospital Cancer Center, Harvard Medical School, Boston, MA USA; 6 BioMed Valley Discoveries Inc., Kansas City, MO USA; 7 Shanghai Hengrui Pharmaceutical Co., Ltd, Shanghai, China; 8 Bioclinica Inc, Princeton, NJ USA; 9 Stanford University, Stanford, CA USA

**Keywords:** Cardiac safety, ECG, Exposure:response modeling, Holter, Oncology, QT, QTc

## Abstract

**Purpose:**

The aim of this analysis was to investigate the potential for ulixertinib (BVD-523) to prolong cardiac repolarization. The mean prolongation of the corrected QT (QTc) interval was predicted at the mean maximum drug concentrations of the recommended phase 2 dose (RP2D; 600 mg BID) and of higher concentrations. In addition, the effect of ulixertinib on other quantitative ECG parameters was assessed.

**Methods:**

In a two-part, phase 1, open-label study in adults with advanced solid tumors, 105 patients [24 in Part 1 (dose escalation) and 81 in Part 2 (cohort expansion)] were included in a QT prolongation analysis. Electrocardiograms (ECGs) extracted from 12-lead Holter monitors, along with time-matched pharmacokinetic blood samples, were collected over 12 h on cycle 1 day 1 and cycle 1 day 15 and analyzed by a core ECG laboratory.

**Results:**

A small increase in heart rate was observed on both study days (up to 5.6 bpm on day 1 and up to 7 bpm on day 15). The estimated mean changes from baseline in the study-specific QTc interval (QTcSS), at the ulixertinib *C*_max_, were − 0.529 ms (90% CI − 6.621, 5.562) on day 1 and − 9.202 ms (90% CI − 22.505, 4.101) on day 15. The concentration: QTc regression slopes were mildly positive but not statistically significant [0.53 (90% CI − 1.343, 2.412) and 1.16 (90% CI − 1.732, 4.042) ms per µg/mL for days 1 and 15, respectively]. Ulixertinib had no meaningful effect on PR or QRS intervals.

**Conclusions:**

Ulixertinib administered to patients with solid tumors at clinically relevant doses has a low risk for QT/QTc prolongation or any other effects on ECG parameters.

**Registration:**

The study is registered at Clinicaltrials.gov (NCT01781429) and was sponsored by BioMed Valley Discoveries.

## Introduction

Ulixertinib is a potent and selective small molecule inhibitor of the extracellular signal-regulated kinases ERK1 and ERK2. Ulixertinib inhibits growth and survival of cancer cells in cultured cell lines, including melanoma, colorectal, and pancreatic cell lines harboring *BRAF* or *RAS* mutations, as well as in animal models. Tumor response was assessed in 101 patients treated with ≥ 600 mg twice daily (BID) ulixertinib, of whom 14 had a partial response per Response Evaluation Criteria in Solid Tumors (RECIST v1.1) criteria [1]. While ulixertinib modestly inhibited (IC_50_, 3.4 µM) the human ether-á-go-go-related gene, it did not significantly prolong the cardiac action potentials recorded from dog Purkinje fibers at concentrations of up to 10 µg/mL. In animal studies, no significant cardiovascular findings were observed with acute (single) oral dosing of ulixertinib of up to 50 mg/kg in dogs [maximum observed concentration (*C*_max_) = 17.3 µM]. In addition, ulixertinib is highly protein bound in multiple species, including human (99.9–100%). Thus, pre-clinical data suggested that ulixertinib would have a favorable cardiac safety profile and low potential for inducing QT/QTc prolongation in patients.

Overall improved efficacy of novel cancer therapies is leading to higher survival rates and a larger population of cancer patient survivorship [2], emphasizing the need for improving drug safety and reducing systemic and organ-specific toxicities of new agents. Commensurate with this progress, recognizing and managing cardiovascular toxicity of cancer therapies are a clinical and regulatory focus. A key safety concern is the potential of drugs, particularly small molecule new chemical entities, to prolong the electrocardiographic (ECG) heart rate corrected QT interval (QTc) and to potentially cause the lethal cardiac arrhythmia Torsades de Pointes [3]. After a series of high profile drug withdrawals and non-approvals in the 1990s and early 2000s, the International Conference on Harmonisation (ICH) adopted the ICH-E14 guidance in May 2005 [4], calling for a methodical assessment of the potential pro-arrhythmia risk of new drugs early in clinical development, typically by conducting a dedicated thorough QT (TQT) study [5].

A key limitation of the ICH-E14 mandated TQT study is that a TQT study is scientifically more robust in healthy volunteers than in patients with serious illnesses, such as cancer patients receiving toxic anticancer drugs [6]. For this reason, and concurrent with recent advancements in the cardiac safety assessment paradigm, which favor QT assessments in routine, early phase drug development clinical trials [7], oncology cardiac safety (QT) assessments are often performed in early phase oncology studies with relatively small patient cohorts. This practice is further supported by the recent update to the ICH E14 guideline, which supports the use of exposure–response (ER) analysis as an alternative to the by-time-point analysis as the primary basis for cardiac safety regulatory decisions [8].

Here, we report on the potential for QT/QTc prolongation effects from a phase 1 oncology study designed to assess the safety, pharmacokinetic (PK), pharmacodynamics (PD), and efficacy of ulixertinib in patients with advanced solid tumors. The present analysis is one of the first to use an advanced Exposure:Response Modeling (ERM) approach, in keeping with recent clinical and regulatory progress in the field. The current ERM strategy is geared toward early phase cardiac safety assessments in small clinical trials, because it uses the full ECG data set in a comprehensive manner. This is especially relevant for oncology cardiac safety studies, which typically lack placebo control, and which often involve small patient cohorts with a large variability in heart rate and QT/QTc interval and patients treated with multiple medications that can introduce challenges for QT assessments and potentially lead to false positive outcomes.

## Materials and methods

### Study design and objectives

The clinical study (Clinicaltrials.gov identifier, NCT01781429) was a first-in-human, two-part, open-label, multicenter phase 1 study designed to assess the safety, PK, and PD of escalating doses of ulixertinib in patients with advanced malignancies. The study was composed of two parts: a dose-escalation phase (Part 1) and a cohort-expansion phase (Part 2). Part 1 established dose-limiting toxicity (DLT), the maximum tolerated dose (MTD), and the preliminary recommended phase 2 dose (RP2D). Part 1 used an accelerated single-patient cohort design, followed by a standard 3 + 3 design, informed by the accrued safety experience throughout the study. Intra-patient dose escalation for patients entering the study at dose levels lower than the RP2D was allowed under specific circumstances. The MTD was defined as the highest dose cohort at which ≤ 33% of patients experienced ulixertinib-related DLTs in the first 21 days of treatment. The RP2D was defined as the MTD and was additionally informed by observations related to PK, PD, and cumulative toxicity observed after multiple cycles. The RP2D was determined to be 600-mg BID during Part 1. In Part 2, patients were initially treated at the preliminary RP2D. Patients received oral doses of ulixertinib BID in 21-day treatment cycles until disease progression, unacceptable toxicity, or another withdrawal criterion was met. Treatment cycles were intended to be administered consecutively without interruption; however, dosing interruptions and/or dose reductions were allowed when necessary to manage toxicities.

ECG data were collected continuously using high-fidelity 12-lead Holter recorders at cycle 1 day 1 and cycle 1 day 15 for 12 ± 2 h during PK sampling. Standard 12-lead ECGs were extracted in triplicates at timepoints corresponding with PK sampling. ECG extraction timepoints were scheduled for cycle 1 day 1 and cycle 1 day 15 at the following timepoints: 0 h (pre-dose) and 0.5, 1 (± 5 min), 2, 4, 6, 8 (± 10 min), and 12 h (± 2 h) post-dose. In Part 1 (dose escalation), ECG data collection was performed for all patients; while in Part 2 (cohort expansion), ECG monitoring was stopped after a sufficient number of patients with valid ECG data were enrolled, based on power calculations using data from Part 1. The current analyses were based on all patients included in Parts 1 and 2 of the study with valid ECG data.

### Patient population

Enrolled patients were men and women ≥ 18 years of age with histologically confirmed metastatic or advanced-stage malignant solid tumors for which no curative therapy existed. Patients had to have an Eastern Cooperative Oncology Group (ECOG) performance status of 0 or 1 and a life expectancy of ≥ 3 months. Other eligibility requirements included adequate cardiac, renal, hepatic, and bone marrow function. Adequate cardiac function was defined as a left ventricular ejection fraction (LVEF) of > 50% [assessed by multi-gated acquisition (MUGA) or echocardiography] and QTc < 470 ms. For Part 2 only, patients with BRAF, NRAS, or MEK mutations who had measurable disease by RECIST were enrolled.

Standard exclusion criteria were applied, including gastrointestinal conditions that could impair absorption of the study drug, and uncontrolled or severe intercurrent or chronic medical conditions. Patients could not take any cancer-directed therapy (e.g., chemotherapy, hormonal therapy, biologic therapy, or immunotherapy) within 28 days or 5 half-lives (whichever was shorter) before the first dose of ulixertinib. A minimum of 10 days was required between termination of any investigational drug and administration of ulixertinib; and any drug-related toxicity, except alopecia, had to have recovered to grade 1 or less. Concurrent therapy with any other investigational agent or drugs known to be strong inhibitors of cytochrome P450 (CYP) enzymes CYP1A2, CYP2D6, or CYP3A4 or strong inducers of CYP3A4 was prohibited.

All patients gave written informed consent before the start of the pre-study examination. The study was conducted according to the protocol and in compliance with ICH Good Clinical Practice guidelines.

### ECG assessments

Digital 12-lead ECGs were recorded continuously using a 12-lead digital Holter recorder (M12R, Global Instrumentation LLC, Manlius, NY, USA) equipped with a removable standard secure digital memory card. The recorder transmitted ECG data continuously, via a Bluetooth connection, to a nearby laptop computer that transmitted the data to the ECG core laboratory (Bioclinica Inc, Princeton, NJ, USA) using a high-speed internet connection.

Standard 12-lead ECGs were extracted automatically from the continuous 12-lead Holter recordings in triplicates at predefined timepoints and analyzed programmatically using an automated algorithm (M12A Enterprise Holter System, Global Instrumentation LLC). All ECGs were manually adjudicated by a board certified cardiologist, and all ECGs of a given patient were read by the same cardiologist. For each 12-lead ECG, three consecutive PQRST complexes and their preceding R–R interval were annotated and measured. Measurements were performed from lead V3 (primary lead), with the aim of obtaining data from the same lead for each patient wherever possible. ECG parameters for any patient and timepoint were considered valid if they were based on at least two readable ECGs of the triplicate.

For QTc determination, two common QT correction (QTc) methods were used. QTcF was calculated using the Fridericia formula (QTcF = QT × RR_s_^1/3^). Correction was performed for each replicate, and the median across replicates was taken for each timepoint. QTcSS, a study-specific QTc, was derived from individual ECGs obtained at the baseline (drug-free) period by fitting a linear mixed-effects model with logQT as the dependent variable, logRR_S_ as the covariate, and a random intercept per patient (QTcSS = QT × RR_s_^−*β*^, where *β* was the regression coefficient of logRR_S_). The estimates of the model parameters were tabulated with two-sided 95% confidence intervals (CIs). In addition, for the pre-dose values, a data set with individual QT, QTcF, QTcSS, and R–R values for each replicate was used to assess the appropriateness of the correction method. QTcF was to be used as the primary correction method if the two-sided 95% CI for *β* included 1/3, the exponent for the Fridericia correction; otherwise, QTcSS was to be considered primary. The appropriateness of the correction methods was investigated as outlined by Tornøe et al. [9].

### ECG analysis sets and validity criteria

ECG parameters for any patient and timepoint were considered valid if they were based on at least two valid replicate ECGs. In addition, two ECG analysis sets were defined as follows: (1) an ECG set included all patients in the safety population who had a valid baseline QTc value and at least one valid post-baseline QTc value. Patients for whom no baseline ECG could be extracted before the PK blood draw for time zero were included if three replicate ECGs could be extracted in the time window between the first PK draw (i.e., baseline) and the first drug administration. Visit 4 (cycle 1 day 15) data were included only if this visit actually took place within 21 days after day 1 (i.e., the day of first drug administration). (2) An extended ECG set consisted of all patients in the ECG set and allowed for patients without a valid pre-treatment baseline ECG to be included if three replicate ECGs could be extracted in the first 15 min after first drug administration. For day 15, this analysis set also included patients irrespective of the actual day the ECG assessments were made, provided the patient received the study drug on that day. In addition, an Exposure:Response (ER) analysis was the intersection of the ECG set and the PK population, as defined in the overall study statistical analysis plan. The ER set was the set of primary interest in this analysis and was used except where specified. The extended ER set was the intersection of the extended ECG set and the PK population. This analysis set was used for robustness analyses.

### Definition of baseline

Baseline was defined as the timepoint of pre-dose assessments on cycle 1 day 1 for each study part. The baseline ECG value was the mean of the triplicate pre-dose ECG values on cycle 1 day 1. For analyses based on the extended ER set, the ECG parameters extracted in the first 15 min after dosing were used as replacement for missing values. Post-baseline ECGs were all ECGs obtained after the defined baseline timepoint.

### QTc analysis endpoints

The analyses were defined in a prospective statistical analysis plan. The primary QTc variable was designated as QTcp. The primary endpoint was the change in QTcp from pre-dose baseline (ΔQTcp). Both QTcF and QTcSS were assessed. Secondary endpoints included the baseline-adjusted effect on heart rate and PR and QRS intervals and the frequency of ECG parameters exceeding a set of given limits. Analyses were performed on the ECG set. Patients for whom no baseline could be extracted before the PK draw at time zero were included if three replicate ECGs were extracted in the time window between the first PK draw (i.e., baseline) and the first drug administration. Day 15 data were only included if the day 15 visit actually took place no later than 21 days after start of treatment.

### PK parameters

For each patient and post-dose QTc value, a corresponding plasma level was calculated by interpolation from the available values. Linear interpolation was used before *T*_max_ (the time to reach *C*_max_), and log-linear interpolation was used after *T*_max_. For times between 0.5- and 6-h post-dose, individual values were excluded if the time between ECG and blood sample collection exceeded 10 min. For later timepoints, this window was increased to 2 h, and extrapolation beyond the last PK measurement was allowed for up to 20 min.

### ER analysis

The primary analysis was based on the primary QTc variable and was performed independently for days 1 and 15 with patients from Parts 1 and 2 pooled. It was based on a linear mixed-effects model with ΔQTcp as the dependent variable, interpolated drug plasma concentration as a continuous covariate, time and part as categorical factors, and a random intercept and slope per patient [10]. In addition, a binary factor indicating what dosing regimen the patient was on was included. For the purpose of this analysis, it was defined prospectively that patients who received an average dose of 300-mg BID or below of ulixertinib were assigned to the low-dose group, and all other patients were included in the high-dose group. In the absence of a placebo group, this factor was introduced as a surrogate for placebo. The degrees of freedom for the model estimates were determined by the Kenward–Roger method [11]. From the model, the slope (i.e., the regression parameter for the concentration) was estimated together with the two-sided 90% CIs and other model parameters. Since time is included as a factor in this model, it allows for the separation of variability attributed to the concentration of the drug from spontaneous diurnal variability; in other words, the estimated time effect can be interpreted as the estimate of diurnal changes independent of drug concentrations and vice versa.

The geometric mean (across patients) *C*_max_ on days 1 and 15 (gm*C*_max_1 and gm*C*_max_15, respectively) of the 600-mg dose was used to estimate the effect of ulixertinib, along with the two-sided 90% CI. The effect estimate was based on the respective primary model as the contrast between patients with the concentration of interest in the high-dose group minus a patient with concentration zero in the low-dose group. For the computation of the CIs, the random nature of *C*_max_ was ignored. Supplementary predictions were performed at 1.5 times the concentration of gm*C*_max_1 and gm*C*_max_15.

Since a joint graphical display of the original ΔQTc and concentration data and the estimated regression line would be difficult, because the influence of time cannot be depicted in a two-dimensional display, we decided to present the regression line for the predicted effect together with the raw ΔQTc data over the concentration. This meant that the regression lines would not necessarily follow the trend seen in the scatter plots. The discrepancy between the two is the effect attributed to time and not to drug concentration.

### Assessment of appropriateness of the primary model

For the above model to be valid, the key assumptions were the absence of hysteresis and the linearity of the concentration-QTc relationship. The former was investigated by a comparison of the time courses of double differences of QTc (ΔΔQTc) and concentration (ΔΔC) (i.e., the difference in mean QTc and mean concentration between the high-dose group and the low-dose group). Since low-dose ulixertinib was administered only in Part 1 of the study, this investigation was done in Part 1 only. Briefly, hysteresis was considered present only if (1) there was a prolongation of more than 5 ms in ΔΔQTc and (2) a delay in the occurrence of the largest prolongation compared with *T*_max_ was > 1 h.

Linearity was tested independently in Parts 1 and 2 by adding a quadratic term to the primary linear model and testing this term. If there was an indication that a linear model was inappropriate in one of the analyses of Parts 1 or 2, alternative non-linear models were to be investigated, and the primary analysis was then to be repeated for the model found to best accommodate the non-linearity detected based on the Akaike information criterion.

### Robustness analyses

To assess robustness of the primary analysis, variants of the analysis were performed. A joint model was fitted on data from both day 1 and day 15. This model was similar to those used in the primary analysis, but had day and interaction day by concentration as additional terms. The term time was defined as time since first dose (i.e., the same times on days 1 and 15 were considered different factor levels). If the interaction term day by concentration was not significant at the two-sided 5% level, a model without this term was also fitted. The primary model for day 1 was also fitted to the extended ER set and the day 15 model was fitted to the extended day 15 ER set. These extended analysis sets included baseline data obtained in the first 15 min after drug administration for cases where no pre-dose value could be obtained; and for day 15, values from those patients where the day 15 visit was actually performed more than 21 days after start of treatment. The primary analysis was also repeated for the correction method (QTcF or QTcSS) that was not used in the primary analysis.

### Categorical analysis of quantitative ECG parameters

Incidences and percentages of patients with the following values were summarized: QTcF values > 450, > 480, and > 500 ms (treatment-emergent); ΔQTcF of > 30 and > 60 ms; PR values > 200 ms, which represent an increase from baseline of at least 25%; QRS values > 110 ms, which represent an increase from baseline of at least 25%; and heart rate decreases > 25% from baseline to a rate of < 50 bpm and increases > 25% from baseline to a rate of > 100 bpm.

### Morphological analysis

Incidence counts for new treatment-emergent morphological abnormalities were also summarized using the following categories for clinical interpretation of the ECGs: normal ECG [sinus rhythm between 50 and 100 bpm with normal P waves, atrioventricular (AV) conduction, QRS complex and ST segment, and T-wave morphologies]; new (i.e., not present at baseline) abnormal ECG findings, probably nonsignificant; abnormal ECG, possibly significant; or unreadable ECG. Any new treatment-emergent repolarization abnormalities (i.e., ST segment abnormalities), T-wave morphology, and AV conduction abnormalities were summarized.

### Combined analysis

Analyses were performed separately for Parts 1 and 2 at the end of each study segment. In this report, we present a joint analysis of the combined Parts 1 and 2 data set, using the methods described above. In addition, although the data reported here include all dose levels, the focus of this report is on the ulixertinib RP2D of 600-mg BID. This is not only because this dose will be the recommended therapeutic dose, but also because this dose group has the largest amount of data since all patients in Part 2 (and the combined data set) initially received this dose (Table 1).

**Table 1:** Patient disposition of the ECG set by dose received, study part, and day

Dose received (BID)	Part 1		Part 2	
	Day 1	Day 15	Day 1	Day 15
Low dose (10–300 mg)	6	5		
High dose (450–900 mg)	18	13		
450 mg			0	1
600 mg			81	54
Total	24	18	81	55

## Results

### Patient enrollment, demographics, and baseline characteristics

A total of 105 patients, a subset of the total number of patients enrolled in this phase 1 study [1], were included in the analysis (Table 1). In Part 1, 24 patients who qualified for the ECG assessment were enrolled; six patients were in the low-dose group. On day 1, all 24 patients were included in the ECG set; while on day 15, only 18 patients (five in the low-dose group) were eligible for inclusion. In Part 2, a total of 85 patients were evaluated for ECG assessment, and 81 were eligible for inclusion in the ECG set. All 81 patients contributed data on day 1, but only 55 had valid data on day 15.

Demographics and baseline characteristics of the patients are provided in Table 2. The most common cancer types were melanoma (37%), colorectal cancer (29%), non-small cell lung cancer (9%), and lung cancer not otherwise specified (6%). All patients had received prior cancer therapy. Most patients (94%) had their tumor molecularly genotyped, and abnormalities (e.g., mutations, fusions, and rearrangements) in *BRAF, KRAS*, and *MEK* were documented. Medical histories included a cardiac disorder in 15% (16 of 105) of patients; these disorders included coronary artery disease, atrial and ventricular fibrillation, tachycardia and bradycardia, aortic valve disorder, pericardial effusion, and pericarditis. In addition, 50% of patients were reported to have a history of hypertension.

**Table 2:** Demographic and baseline characteristics

Parameter	Total (*n* = 105)
Age (years), mean (SD)	59.1 (12.43)
Sex, *n* (%)	
Female	42 (40)
Male	63 (60)
Race, *n* (%)	
White	90 (86)
Black African Heritage or African American	6 (6)
Asian	5 (5)
Other	4 (4)
BMI (kg/m^2^), mean (SD)	27.32 (5.93)
Baseline ECOG performance status, *n* (%)	
0	37 (35)
1	67 (64)
2	1 (< 1)
Cancer type, *n* (%)	
Melanoma	39 (37)
Colorectal	20 (19)
NSCLC	9 (9)
Lung (NOS)	6 (6)
Other cancers (e.g., glioblastoma, thyroid, prostate, gastrointestinal, pancreatic, salivary gland, squamous cell carcinoma, etc)	31 (29)
LVEF (%)	*n* = 104 (> 99)
Assessed by echocardiogram^a^, *n* (%)	99 (94)
Assessed by MUGA scan^a^, *n* (%)	5 (5)
Mean LVEF (SD)	62.15 (6.243)
Median LVEF	62.8
Minimum, maximum LVEF	43.0, 82.0

*ECOG* Eastern Cooperative Oncology Group, *LVEF* left ventricular ejection fraction, *MUGA* multigated acquisition, *NOS* not otherwise specified, *NSCLC* non-small cell lung cancer, *SD* standard deviation

^a^Echocardiogram and MUGA were not performed on one patient at the baseline evaluation

### Selection and validation of primary endpoint variable

Consistent with the statistical analysis plan, the primary QTc endpoint parameter was tested and validated before commencing analysis. The estimate of the QTcSS model parameter (based on the individual replicates) yielded a slope of 0.39 ms/s with a two-sided 95% CI of 0.348–0.422 ms/s. Since these CIs do not include 0.333, which characterizes the Fridericia correction, the QTcSS was selected as the primary correction method for this analysis.

A linear regression of QTcF and QTcSS on the R–R interval (in seconds) further supported the superiority of QTcSS by showing a regression coefficient closer to zero (10.2 vs 37.2 ms/s for QTcF). Likewise, the RMSS for QTcSS was slightly smaller than that for QTcF (18.6 vs 19.1 ms/s). Consequently, QTcSS was confirmed as the primary endpoint variable for this analysis.

### Exclusion of individual measurements and interpolation of PK values

PK values obtained at the time of ECG samplings were interpolated from the available PK data as explained above (see “PK parameters”). Overall, 84 measurements, i.e., 6% of all measurements, had to be excluded because of violation of time constraints.

### Appropriateness of model

In Part 1, ΔΔQTcSS and ΔΔQTcF, the mean time-matched differences in QTc between the high- and low-dose groups, indicated a QTc shortening rather than a prolongation. Consequently, hysteresis was considered inconsequential. Likewise, there was no indication that the linear model was inappropriate.

### Primary analysis

**Fig. 1:**
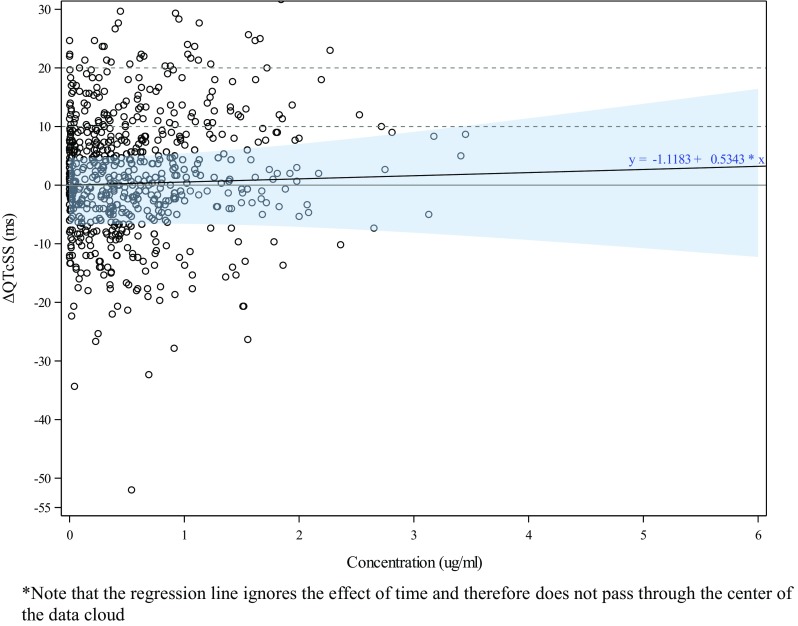
Primary model: raw ΔQTCSS values vs concentration on day 1

**Fig. 2:**
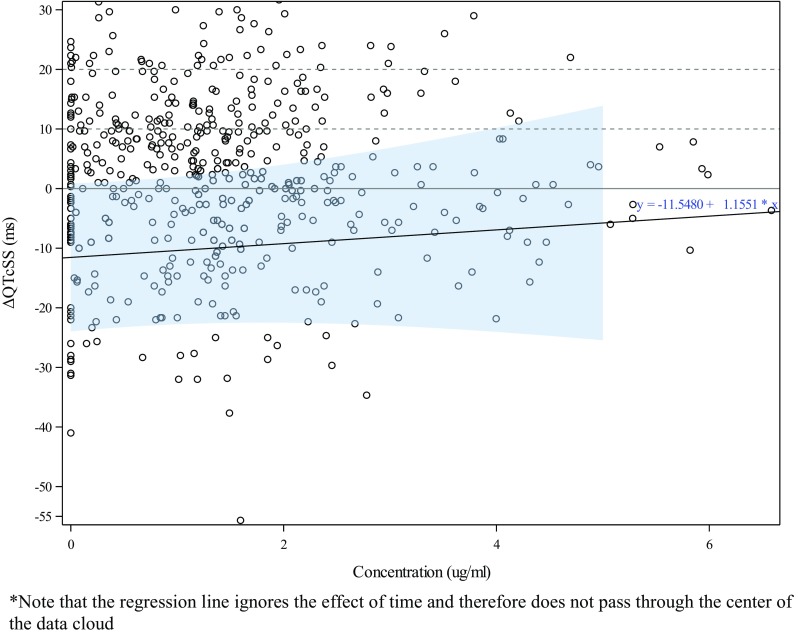
Primary model: raw ΔQTCSS values vs concentration on day 15

The primary models showed positive, although non-significant regression slopes, for the relationship between ulixertinib concentration and QTcSS (0.53 and 1.16 ms per µg/mL for days 1 and 15, respectively) (Table 3; Fig. 1 for day 1 and Fig. 2 for day 15). The standard error for the slope estimates was comparable in both models, but the standard errors for all other parameters, including the estimates for the time effect not attributable to the drug (Table 3) were substantially larger for day 15. In both models, the parameter for Part was not significant; for this reason, pooling of the two parts of the study seemed appropriate. The parameter for dose group was small and not significant for day 1. However, for day 15, it was substantially larger and with *p* = 0.068, and it was significant at the two-sided 10% level but not significantly different from zero at the two-sided 5% level because of the larger standard error. Since the low-dose group was used as a surrogate for placebo in this analysis, the parameter for dose group was an indicator for the appropriateness of the linear model used. For day 1, this parameter did not give any indication that the model was inappropriate. For day 15, the result was less clear, but if a two-sided *p* value of 5% was taken as the criterion, a linear model without hysteresis appeared acceptable.

**Table 3:** Key parameters of the primary models

Day	Parameter	Estimate	SE	DF	*t* value	*p* value	90% CI
1	C:E slope	0.53	1.14	506	0.469	0.639	(− 1.343, 2.412)
	Dose group^a^	− 1.12	2.88	171	− 0.388	0.698	(− 5.880, 3.643)
	Part	− 0.56	1.68	211	− 0.331	0.741	(− 3.327, 2.215)
15	C:E slope	1.16	1.74	109	0.664	0.508	(− 1.732, 4.042)
	Dose group^a^	− 11.55	6.24	90.9	− 1.849	0.068	(− 21.925, − 1.171)
	Part	6.68	4.07	105	1.642	0.104	(− 0.072, 13.430)

Model used dQTcSS ~ *C* + dg + time + part + (1 + *C*|subj*P*)

Kenward–Roger approximation was used

*C:E slope* concentration:effect (QTc) regression slope, *CI* confidence interval, *DF* degrees of freedom, *SE* standard error

^a^The parameter for dose group replaces the intercept in a model with time as factor. It is an indicator for the appropriateness of the model, with values significantly different from zero indicating misfit

### Predictions of QTc effect based on the primary models

The predicted QTcSS change from baseline values at the days 1 and 15 *C*_max_ were both negative (− 0.529 and − 9.202 ms, respectively), with both two-sided 90% CIs well below the 10 ms regulatory threshold (5.562 and 4.101 ms for days 1 and 15, respectively). The predictions for higher exposures at 1.5 times the observed *C*_max_ were also below 5 ms for both days, and the 90% CIs were well below 10 ms for days 1 and 15 (6.401 and 6.977 ms, respectively). The wider CIs on day 15 were attributable to the large variability of this prediction on that study day (Table 4).

**Table 4:** Predicted QTcSS effect based on the primary models

Day	Dose/condition	Concentration (µg/mL)	Prediction (ms)	90% CI
1	*C*_max_	1.102	− 0.529	(− 6.621, 5.562)
	1.5*C*_max_	1.652	− 0.236	(− 6.873, 6.401)
15	*C*_max_	2.031	− 9.202	(− 22.505, 4.101)
	1.5*C*_max_	3.046	− 8.030	(− 23.036, 6.977)

Model used dQTcSS ~ *C* + dg + time + part + (1 + *C*|subj*P*)

Consequently, the predictions based on the two models (for days 1 and 15) excluded a QTc effect of concern on both days for concentrations associated with ulixertinib doses of 600 mg and above. The width of the CI on day 15 indicated that, in this group of cancer patients, a prediction of the drug effect after 15 days of treatment is quite uncertain; however, given a terminal phase elimination rate of approximately 9–11 h, ulixertinib is most likely at steady state by day 15. Thus, a further increase in the QTc under the same conditions is quite unlikely. Furthermore, PK analyses of samples collected after day 15 confirm that steady state was achieved by day 15. Even under these conditions, a relevant QTc prolongation of concern can be excluded.

### Sensitivity and robustness analyses

To further corroborate the model and investigate the robustness of its outcomes, we performed a number of sensitivity analyses, including fitting a joint model for the day 1 and day 15 data, refitting the joint model for day 1 and day 15 without the interaction term, and fitting the primary model to QTcF. Separate analyses of the two parts of the study were also performed (data not shown).

A joint model combining days 1 and 15 was performed as a sensitivity analysis and produced an estimated concentration-QTc slope that was nonsignificant and negative [− 4.9 ms (90% CI − 11.2, 1.4) for day 1 and − 3.6 ms (− 10.529, 3.328) for day 15]. As a result, the predictions and CIs were also smaller. However, as pointed out above, the smaller standard error of the slope estimates, which resulted in substantially lower upper limits of the CI for the predictions, should be interpreted with caution because of the observed differences in variability between the two study days. Furthermore, the results using QTcF did not differ substantially from those obtained with QTcSS. It should be noted that the slope on day 15 was somewhat larger when using QTcF, but still not statistically significant. The predicted QTcF at the day 15 *C*_max_ (− 15.012 ms) was even more negative than that seen with QTcSS and reached statistical significance. Likewise, the upper limit of the CI for this prediction was − 1.928 ms compared with 4.101 ms for QTcSS.

### Effects on other ECG parameters

For the purpose of summarizing the ECG parameters, data from the high-dose group (600 and 900 mg), which was primarily the 600-mg group (Table 1), are presented. The mean heart rate at baseline was 75.8 bpm for the high-dose group. Change from baseline data for this group showed a small increase in heart rate during the 12-h monitoring, up to 5.6 bpm on day 1 (at 4-h post-dose) and up to 7 bpm on day 15 (at 6- and 12-h post-dose) (Table 5). The mean PR interval at baseline was 164.3 ms for the high-dose group. Change from baseline data showed a small decrease in the PR interval on day 1 of up to 1.8 ms (at 12-h post-dose) and a small increase of up to 3.7 ms on day 15 (at 1-h post-dose), followed by a small decrease thereafter. Mean QRS data were quite stable on both days with small increases in QRS of up to 0.8 and 3.1 ms on days 1 and 15, respectively.

**Table 5:** Mean (SD) change from baseline in ECG parameters (high-dose group)

Study day	ECG timepoint	HR (bpm)	PR (ms)	QRS (ms)	QT (ms)	QTcSS (ms)	QTcF (ms)
Day 1	0.5 h post-dose	− 2.1 (4.8)	2.6 (13.3)	0.4 (4.9)	5.2 (10.9)	1.3 (8.9)	1.9 (8.5)
	1 h post-dose	− 0.9 (6.0)	1.1 (8.7)	0.8 (5.0)	3.7 (12.5)	2.4 (9.4)	2.6 (8.8)
	2 h post-dose	1.5 (7.3)	− 0.4 (8.5)	0.6 (4.7)	1.2 (16.5)	4.6 (9.5)	4.2 (9.5)
	4 h post-dose	5.6 (8.7)	− 1.2 (9.5)	0.8 (5.2)	− 7.5 (20.6)	3.4 (12.2)	1.8 (12.5)
	6 h post-dose	4.4 (9.4)	− 0.9 (18.0)	0.0 (5.4)	− 7.1 (22.9)	1.2 (12.6)	− 0.0 (13.3)
	8 h post-dose	4.8 (8.6)	− 0.8 (16.1)	0.5 (5.7)	− 8.5 (20.5)	1.2 (11.0)	− 0.2 (11.4)
	12 h post-dose	4.8 (10.0)	− 1.8 (13.6)	0.1 (5.6)	− 10.3 (25.0)	− 1.1 (12.6)	− 2.5 (13.4)
Day 15	prior to dosing	3.2 (13.5)	2.1 (11.5)	0.6 (6.7)	− 8.0 (34.0)	− 2.3 (17.0)	− 3.1 (18.1)
	0.5 h post-dose	1.2 (11.5)	2.9 (13.7)	3.1 (5.9)	− 0.6 (31.5)	2.0 (16.0)	1.7 (17.2)
	1 h post-dose	− 0.9 (6.0)	3.7 (14.3)	1.9 (6.0)	1.4 (30.5)	4.9 (16.6)	4.5 (17.3)
	2 h post-dose	3.8 (11.7)	2.0 (15.1)	1.6 (5.9)	− 2.1 (29.7)	6.3 (15.1)	5.2 (15.7)
	4 h post-dose	6.1 (11.2)	2.9 (12.3)	1.7 (6.6)	− 9.2 (29.1)	3.1 (16.9)	1.4 (17.5)
	6 h post-dose	7.0 (12.9)	− 2.1 (14.1)	1.3 (5.9)	− 11.6 (31.7)	1.8 (14.5)	− 0.1 (16.0)
	8 h post-dose	6.5 (12.9)	− 0.9 (12.8)	1.5 (6.0)	− 10.6 (31.5)	2.2 (14.4)	0.4 (15.8)
	12 h post-dose	7.0 (12.2)	− 1.7 (13.0)	2.1 (7.0)	− 13.0 (31.8)	0.4 (15.3)	− 1.5 (16.7)

*N* = up to 99

High–dose = 600–900 mg

*Bpm* beats per minute, *HR* heart rate, *ms* millisecond, *SD* standard deviation

Uncorrected QT interval data largely showed reductions of up to 10.3 ms on day 1 and 13.0 ms on day 15 (both at 12-h post-dose). Mean QTcSS values showed trivial changes of up to 4.6 and 6.3 ms on days 1 and 15, respectively (both at 2-h post-dose). QTcF mean values followed a similar trend with maximum values of 4.2 and 5.2 ms on days 1 and 15, respectively, (again, at 2-h post-dose for both days).

### Categorical analyses

The number of patients exceeding the predefined QTcF thresholds (450, 480, and 500 ms) at any time was generally small and mostly confined to the lower threshold of > 450 ms [seven of 88 patients (8%) had a QTcF value > 450 ms, one had a value > 480 ms, and one had a value > 500 ms]. The changes from baseline in QTcF (> 30 or > 60 ms) were also small, primarily at the lower level (> 30 ms), and more frequently on day 15 (7 vs 3%). No patient had a change from baseline of > 60 ms on day 1, and only one patient reached this value at day 15.

Heart rate changes were primarily positive [i.e., increases above the predefined thresholds (> 100 bpm and Δ% > 25%)], quite frequent (47% at any time), and occurred equally on both study days. Reductions in heart rate (< 50 bpm and Δ% < − 25%) were observed in 8% of patients, primarily on day 1. Overall, these data support the findings of the summary statistics, suggesting a small increase in heart rate.

Changes in PR values above the threshold of 200 ms and ΔPR > 25% were observed in only one patient on day 1. Considered in conjunction with the central tendency analysis, these changes are not clinically meaningful. No QRS prolongation meeting the predefined criteria (> 110 ms and ΔQRS > 25%) were observed.

Summaries of the overall ECG diagnostic classification showed no relevant change from the baseline distribution (53, 11, and 34% for normal, insignificantly abnormal, and significantly abnormal, respectively) on day 1. However, on day 15 an increase in the proportion of significantly abnormal ECGs was observed (42, 2, and 56%, respectively) 12-h post-dose. These observations need to be considered in the context of the advanced illness of the patient population; thus, ECG changes are to be expected.

### Morphological analyses

Repolarization abnormalities, comprised of ST segment and T wave and U wave changes, including events of QT prolongation (> 450 ms) or QT shortening (< 340 ms), were reported in similar proportions of patients at baseline (5%) and post-baseline (up to 7%). Likewise, T wave morphology changes, including biphasic, flat, inverted, notched, and peaked T waves, were similarly reported at baseline (15%) and post-baseline timepoints (up to 18%). Conduction abnormalities, consisting of all the types of AV blocks (although no Mobitz 2 or complete heart block events were observed), were reported in 10% of patients at baseline and up to 13% post-baseline.

## Discussion

Analysis of cardiac repolarization (QT/QTc) data from oncology drug development studies is particularly challenging because of study design limitations, patients’ conditions, and a range of confounding factors. Oncology studies usually do not include a placebo control group, may have a limited PK exposure due to drug toxicity, may be underpowered for QT/QTc assessment, and are often conducted in busy hospital oncology departments or outpatient clinics less experienced with the collection of high-quality ECG data. Moreover, cancer patients often have serious medical conditions and are susceptible to clinical deterioration due to disease progression and treatment-related complications, including multi-organ drug toxicities, intercurrent infections, and fluid and electrolyte disturbances [6]. In addition, they may have had prior exposure to drugs associated with cardiotoxic effects and are likely to be treated with multi-drug combinations with known or potential cardiac adverse effects, including QT/QTc interval prolongation and possibly other ECG and cardiovascular effects. All of the above may lead to higher variability in heart rate and the QT/QTc interval, further undermining the ability to rule-in or rule-out a relevant QT/QTc effect. For all of these reasons, the current ICH-E14 paradigm is a challenge when applied to oncologic agents, where healthy volunteer studies are not possible. As a consequence, recent changes to the ICH-E14 guidance promoting the use of ERM analysis in early phase studies, present a unique opportunity for improved QT analysis in oncology drug development.

In the current study, we used advanced ERM in an early phase oncology study. Although this method lacks placebo data [12], it is nevertheless expected to be more powerful than the traditional by-timepoint analysis, and promising results have also been obtained without the availability of placebo data [13]. Moreover, the use of the low-dose group as a surrogate for placebo should further enhance the value of this method.

Selection of the most appropriate QTc endpoint variable for a given study population may have an impact on estimating the QT/QTc effect of a drug, especially if there is a concurrent effect on heart rate [14]. For this purpose, we investigated the relationship between the raw (uncorrected) QT and R–R intervals during drug-free study periods. The resulting drug-free QT:RR regression slope, estimated at 0.39 ms/s (90% CI 0.348, 0.422 ms/s), was then used as the power exponent for the QTcSS. This population-specific QT:RR slope was somewhat higher than the Fridericia (QTcF) exponent (0.33) and was shown to be a superior correction method for this data set. However, since the observed effect on heart rate was relatively small, no meaningful differences between the correction methods were observed.

### Effect on ECG parameters

Analysis of the heart rate data, using descriptive statistics, suggests a small increase in heart rate of up to 6 and 7 bpm on days 1 and 15 at 4 and 6-h post-dose, respectively.

The appropriateness of the ER model developed for this study to assess the relationship between ulixertinib exposure (concentrations) and QTc effect was thoroughly investigated and confirmed following the predefined statistical analysis plan. Tests for linearity and hysteresis (delayed effect) confirmed that a linear model was appropriate and that a correction for hysteresis was not required. Sensitivity analyses to examine the robustness of the model and the selected primary QTc variable were also performed and confirmed the fitness of the model parameters and predictions.

On the basis of the primary model, the predicted QTcSS effect at the day 1 estimated *C*_max_ (1.102 µg/mL) showed a small mean negative change of − 0.529 ms (i.e., QTc shortening) with an upper 90% CI of 5.562 ms (i.e., worst-case scenario QTc prolongation). The predicted mean QTcSS change for day 15 at the estimated *C*_max_ (2.031 µg/mL) showed a larger mean shortening of − 9.202 ms with an upper CI of 4.101 ms. The wide CI on day 15 (− 22.505, 4.101) reflects, in part, the expected wide variability in this severely ill population of oncology patients [5], although it may also indicate a progressive change in patients’ condition during the study or, possibly, a deterioration in data quality.

The primary analysis, designed to separate the effect of drug concentration from diurnal and ultra-diurnal effects not attributable to the drug concentration, yielded a nonsignificant positive slope of about 0.53 ms per µg/mL. The predicted diurnal variation of the mean QTcSS interval (independent of drug concentration) showed an estimated peak effect of 4.88 ms at 2-h post-dose on day 1 and 10.87 ms at 2-h post-dose on day 15, with corresponding upper CIs of 9.196 and 18.426 ms, respectively. Notably, the estimated QTc values related to diurnal variability are much larger than the predicted drug effects, emphasizing the relatively small magnitude of drug effect, if any, on the QTc interval (noting that both slopes were not significantly different from zero).

Overall, these findings are consistent with a favorable repolarization profile of ulixertinib. No relevant changes in cardiac conduction (PR and QRS intervals) or repolarization morphology (ST segment and T wave) were observed in this study. A small negative mean QTc effect that, at the expected therapeutic exposures, produced mean changes below 5 ms and upper 90% CIs that were well below the 10-ms regulatory threshold for non-oncologic drugs and well below the commonly used 20-ms threshold for oncologic medications. The QTc data analyzed with ulixertinib in this single-agent study support its further development, and its metabolism by multiple metabolic pathways suggests a small risk for higher drug exposures due to drug interactions or metabolic inhibition.

### Study limitations and data interpretation

Similar to other oncology studies, the main limitations of this phase 1 oncology study was the absence of a placebo control group and thus the ability to adjust for disease progression and have a direct correction for diurnal variability. In addition, the large variability of the QT/QTc intervals, particularly on day 15, is a challenge commonly observed in oncologic studies. In the present analysis, the absence of a placebo group was compensated for by using the low-dose groups as surrogate for placebo. This allowed an estimation of a dose group effect that was shown to be a sensitive indicator for the appropriateness of the model. However, it must be kept in mind that the low-dose group consisted of only six patients. In addition, a possible change in the patients’ conditions during the first 2 weeks of the study, resulting in the observed higher variability in the QTc data on day 15, cannot be excluded. This is also reflected in the larger standard error for all parameter estimates, except for the slope parameter for this day.

Compared with the analysis based on day 1, results based on day 15 seem to be more prone to errors, as reflected by the large width of the CIs for the predictions. Considering this caveat, reassuringly the predictions for day 15 consistently excluded a QTc prolongation of concern (i.e., 10 ms and above).

While this dose group effect was small and non-significant for day 1, it was larger for day 15. Although with a *p* value of 0.068, it misses the threshold where the model would be considered inadequate, the moderate fit of the day 15 model somewhat limits the value of the predictions for this day. As pointed out earlier, such a limitation is not unexpected in a study in oncologic patients.

The totality of the data, including those collected from patients in the dose-escalation and early cohort-expansion phases, along with statistical power considerations, led to a decision by the ulixertinib team to discontinue collection of additional Holter data from patients enrolled at the end of cohort expansion.

## Conclusion

On the basis of the analysis of the ECG data from this phase 1 oncology study in patients with solid tumors treated with ulixertinib administered at clinically relevant doses, primarily at the RP2D of 600-mg BID, ulixertinib has a low risk for QT/QTc prolongation.
